# Concordance of self-reported and medical chart information on cancer diagnosis and treatment

**DOI:** 10.1186/1471-2288-11-72

**Published:** 2011-05-18

**Authors:** Vinay Gupta, Kai Gu, Zhi Chen, Wei Lu, Xiao Ou Shu, Ying Zheng

**Affiliations:** 1 Department of Cancer Control & Prevention, Shanghai Municipal Center for Disease Control and Prevention, 1380 Zhongshan Road West, Shanghai 200336, China; 2 College of Physicians and Surgeons, Columbia University, 630 West 168th Street, New York, NY 10032, USA; 3 Division of Epidemiology, Vanderbilt Epidemiology Center, Department of Medicine, Vanderbilt University Medical Center, 2525 West End Avenue, Suite 600, Nashville, TN 37203, USA

## Abstract

**Background:**

Self-reported information is an important tool for collecting clinical information for epidemiologic studies and in clinical settings where electronic medical records are not employed and shared.

**Methods:**

Using data collected from the Shanghai Breast Cancer Survival Study (SBCSS), a population-based, prospective cohort study of 5,042 women diagnosed with breast cancer in Shanghai, China, we compared the concordance of patient questionnaire responses to a survey administered approximately 6 months after cancer diagnosis with medical chart information obtained from the diagnostic hospitals for several disease and treatment-related variables.

**Results:**

Of 5,042 SBCSS participants, medical chart information was available for 4,948 women (98.1%). Concordance between patient self-reported and medical chart information was high for the majority of disease-related variables, including: diagnosing hospital (agreement: 98.7%, kappa: 0.99), type of surgery conducted (94.0%, 0.53), ER/PR status (94.5%, 0.91), and tumor position (98.2%, 0.97), as well as for important calendar dates, such as date of diagnosis, surgery, and first chemotherapy treatment. The 10 most commonly used chemotherapeutic drugs were all reported with agreement rates of at least 82%, with associated kappa values that ranged from 0.41 for calcium folinate to 0.76 for vinorelbine.

**Conclusions:**

Our study found high validity for patient self-reported information for a variety of disease and treatment-related variables, suggesting the utility of self-reports as an important source of clinical information for both epidemiological research and patient care.

## Background

Epidemiologic studies monitoring clinical information often rely on data gathered from patient self-reports. However, not only do self-report instruments such as interviews or questionnaires depend on adequate patient comprehension and recall ability, they also are affected by the wording of questions, the length of recall required, and the interview setting [1-4]. Measuring the accuracy of self-reported information is only possible if the derived data have also been recorded in parallel by a so-called gold standard such as, arguably, a medical chart [5]. Studies across several medical specialties have employed this strategy to validate their respective patient-reported data [6-10]. However, the vast majority of such studies analyze medical systems in Western or otherwise highly developed nations, where electronic medical records or other methods of optimized information sharing are generally available.

Although the investment required to abstract pertinent medical record information on a broader scale can be prohibitive [11], the benefits of validating patient self-reporting techniques are numerous. Misclassifications in exposure and outcome assessments could result in biased risk assessments and a substantial loss of statistical power in epidemiologic studies. The accuracy of patient-reported information would also greatly assist in decisions regarding treatment plans and clinical research [12,13], particularly in developing countries where medical record information is often not computerized or shared among care providers.

The primary objective of the study is to provide a comprehensive analysis evaluating the concordance of self-reported data with medical chart information on cancer diagnosis and treatment information among a cohort of 5,042 breast cancer patients living in Shanghai, China. This study provides information on the utility of self-reported medical information for use in large-scale epidemiology studies of cancer outcomes and as a means of sharing medical information in a developing nation whose citizens routinely access multiple hospitals for their care without inter-institution information sharing or a well-established electronic medical records infrastructure.

## Methods

### Study population

Study subjects for this analysis are participants of the Shanghai Breast Cancer Survival Study (SBCSS), a large, population-based, prospective cohort study of 5,042 women who were diagnosed with breast cancer between March 2002 and April 2006 and were permanent residents of Shanghai, China. Details about the SBCSS have been described previously [14]. In brief, patients were identified from the population-based Shanghai Cancer Registry and recruited into the study approximately 6 months after cancer diagnosis. Of the 6,299 identified cases, 5,042 provided written, informed consent and participated in the study (participation rate: 80.0%). For the remaining cases, 757 (12.0%) refused to participate, 258 (4.1%) were absent during study enrollment, 83 (1.3%) could not be contacted, and 159 (2.5%) were excluded for other miscellaneous reasons such as health or communication problems.

This study was approved by the institutional review boards of Vanderbilt University Medical Center and the Shanghai Municipal Center for Disease Control and Prevention.

### Data collection

Nine trained interviewers, all of them retired medical professionals, visited study participants' homes and conducted an in-person interview in which they administered the study questionnaire approximately 6 months after cancer diagnosis. The mean time for each interview was 67 minutes (standard deviation: 16 minutes). Medical charts from the time period in which the initial breast cancer diagnosis occurred were reviewed and pertinent information was abstracted using structured questionnaires. Medical charts were abstracted by 55 tumor registrars at the hospitals that cared for our study participants (over 90% of participants were seen by 31 major hospitals in Shanghai). Interviewers and tumor registrars completed separate, rigorous training programs and were instructed to follow standardized study protocols. Participants' medical chart information was not available to the interviewers who conducted the in-person interviews. We also implemented a quality control initiative in which all information abstracted from the medical charts was reviewed by the staff of the Shanghai Municipal Center for Disease Control and Prevention, Department of Cancer Prevention; errors were identified and corrected individually by consulting the registrars at the appropriate hospital.

Information gathered about disease and treatment-related factors from the questionnaire included: hospital of diagnosis, occurrence of corrective surgery, dates of diagnosis and surgery, tumor position, estrogen receptor (ER) and progesterone receptor (PR) status, surgical procedures conducted, and chemotherapy or radiotherapy regimens employed up to approximately 6 months after cancer diagnosis.

Using a structured questionnaire, tumor registrars collected cancer- and treatment-related information from each patient's medical chart from the diagnostic hospitals where patients typically received their initial cancer treatment. Information gathered included: hospital of diagnosis, date of breast cancer diagnosis, date of surgery, stage of tumor-node metastasis (TNM) at the time of primary diagnosis, ER/PR status, types of surgical procedures (e.g., partial versus radical mastectomy), and chemotherapy or radiotherapy use. The time period covered by the medical chart varied from 1 to 3 months. The reason for this variation was because medical chart reviews only took place at each patient's respective diagnostic hospital and tumor registrars were instructed only to collect information related to cancer diagnoses and the first course of chemotherapeutic regimens. In Shanghai, it is not uncommon for cancer patients to receive initial cancer treatment at one hospital and later switch to other hospitals for further adjuvant treatments. The decision to limit medical chart abstraction to the first treatment regimen was partly due to practical considerations.

### Statistical analysis

The focus of our validation study centered on the concordance of patient self-reported data with medical chart information regarding disease- and treatment-related variables. Specifically, we analyzed 3 aspects of the concordance of patient responses with medical chart data: (1) recall of important diagnostic parameters of disease, (2) recall of the occurrence and timing of important disease-related events, and (3) recall of the use of the 10 most commonly prescribed chemotherapeutic agents for breast cancer treatment in our cohort. Tumor position was coded as left breast, right breast, or both, and most dates were formatted to either the exact date in Julian code (date coded as the raw number of days from a predetermined baseline of July 1st 1960 as day 0) or as a six-digit code (YYYYMM), which was interpreted manually.

The Kolmogorov-Smirnov D test was used to test data for normality. The Mann-Whitney rank sum test was used to compare differences in the median age at diagnosis between patients who were included in the study and those who were excluded. Fisher's exact test was used to compare differences among categorical variables. Logistic regression analysis was conducted to determine whether demographic characteristics affected concordance among disease- and treatment-related variables. The significance levels were set at *P *< 0.05 for two-sided analyses. Medical chart information was used as the gold standard to determine the validity of self-reported information. Within the current study's context, variables evaluated included: diagnosing hospital, queries regarding whether an operation was conducted, the type of operation performed, ER/PR status, and anatomic position of the tumor (left/right/both breasts). Measures of concordance used were Cohen's kappa statistic and percent agreement. Prevalence- and bias-adjusted kappa was used where the prevalence of a response was zero (15). The kappa statistic measures the extent of exact agreement, adjusting for chance agreement with values greater than 0.75 representing excellent concordance; values of 0.40 to 0.75 representing moderate concordance; and values less than 0.40 representing poor concordance [15]. All statistical analysis was performed using STATA SE version 11 (StataCorp., College Station, Texas).

## Results

Of 5,042 SBCSS participants, medical chart information was available for 4,948 (98.1%). The age range of our study participants was 20.5 to 75.0 years at diagnosis (mean: 53.5 years). Participants with medical chart information were younger than those without it (median of 51.1 years vs. median of 54.2 years, *P *< 0.03), but the two patient groups were otherwise similar regarding socio-demographic variables such as education level and per capita income. In addition, they had similar self-reported treatment-related characteristics (Table 1). Among our study participants (n = 4,948), 33.95% had stage I (n = 1,680), 33.25% stage IIa (n = 1,645), 16.92% stage IIb (n = 837), 9.42% stage III (n = 466), and 0.49% stage IV (n = 24) breast cancer. Disease stage information was missing for 5.98% of study participants (n = 296). All baseline characteristics reported above were drawn from patients' medical charts.

**Table 1:** Baseline Characteristics of Study Participants and Excluded Cases, Shanghai, China, 2002-2006.

Characteristic	Study cohort (n = 4,948)	Excluded cases^a ^(n = 94)	*P*-value
Median age at diagnosis, (IQR) ^b^	51.1 (46.4 - 60.5)	55.2 (47.5-66.9)	<0.02
Monthly income (yuan) (%)			
<500			0.97
500-699	11.1	9.6	
700-999	16.5	14.9	
1000-1999	29.4	32.0	
>2000	31.0	32.0	
	12.0	11.7	
Education level (%)			0.20
<High school	46.4	45.7	
High school	37.6	37.2	
>High school	16.0	17.1	
Received chemotherapy (%)	91.2	89.4	0.47
Received radiotherapy (%)	32.1	33.0	0.83

^a^Excluded cases represent women enrolled in the Shanghai Breast Cancer Survival Study who had missing patient questionnaire data.

^b^IQR: Inter-quartile range.

The time-gap between the collection of the in-person interview and the date of diagnosis was a median of 189 days (interquartile range (IQR): 177-202 days), or approximately 6.5 months. Date of diagnosis was defined as the day on which a surgically or biopsy-confirmed tissue diagnosis of malignancy was obtained. Conversely, the time period covered by the information abstracted from medical charts was a median of 2 months (IQR: 1-3 months) after cancer diagnosis. Medical chart data showed that patients generally began chemotherapeutic regimens at a median of 1 month post-diagnosis (IQR: 1-2 months) and that they typically completed these regimens within a median of 1 month (IQR: 1-2 months). Patient self-reports represented a median of 127 additional days worth of disease-related information compared with the medical chart.

### Concordance in reporting of diagnostic parameters of disease

The agreement between both survey instruments on reporting the correct hospital of diagnosis (98.7%), whether or not curative surgery was performed (99.4%), ER/PR status (94%), tumor position (98%), and type of surgery (94.0%) was high. Kappa statistics were above 0.9 for all variables except for type of surgery, indicating excellent concordance. The kappa statistic for type of surgery was 0.53, indicating moderate concordance (Table 2).

**Table 2:** Concordance of Patient Responses with Medical Chart Data when Examining Diagnostic Parameters of Disease, Shanghai, China, 2002-2006.

Disease characteristic	Number of observations	^a^Agreement (%)	Kappa	Sensitivity % (95% CI)	Specificity % (95% CI)
Diagnosing hospital	4,943	98.7	0.990	-	-
Did you receive an operation? (Yes/No)	4,948	99.4	0.900^b^	100.0 (99.9-100.0)	3.2 (0.1-16.7)
Type of surgery conducted	4,917	94.0	0.530	-	-
ER/PR ^c ^status	3,637	94.5	0.910	-	-
Tumor position	4,948	98.2	0.970	-	-

^a ^Percentage of women reporting the exact same response for a given question across both surveys.

^b ^Prevalence- and bias-adjusted Kappa.

^c ^ER: Estrogen receptor; PR: Progesterone receptor

### Concordance in reporting the occurrence of disease-related events

Since the patient survey covered a longer period of time than the medical chart questionnaire, agreement and concordance rates for disease- and treatment-related information were calculated only among patients who had responses observed in both assessment tools. Patient recall of the occurrence and timing of important disease-related events was generally in high agreement with corresponding medical chart data (Table 3). The concordance in patient reporting of important dates (to the exact month and year), such as the date of diagnosis of breast cancer (agreement: 93.5%) or date of surgery (agreement: 86.4%), was high. In addition, patients were able to accurately recall whether or not they received chemotherapy or radiotherapy and also provide accurate time frames for these courses of treatment, as relevant agreement values were over 79%. Usage rates for each of these treatment modalities was consistently higher as reported by the patient questionnaire relative to the medical chart, which is likely related to the patient questionnaire covering a longer period of time. Further, in most cases, patients were able to report the dates they received these treatments to within 3 days of the date recorded by their medical chart. Specifically, the mean difference in days between the dates of initiation of radiotherapy and chemotherapy as measured by both modalities was 2.8 and 1.6, respectively. These findings suggest that, despite the nearly 4-month time lag, patients were able to recall therapy time frames with excellent accuracy.

**Table 3:** Concordance in Reporting the Occurrence and Timing of Important Disease-Related Events from Patient Responses and Medical Chart Data, Shanghai, China, 2002-2006.

Name of event ^a^	% reported in PQ^b^	% reported in MC^c^	Number of shared observations ^d^	Agreement (%)	Kappa	Sensitivity % (95% CI)	Specificity % (95% CI)
Date of diagnosis of breast cancer	100.0	99.4	4,916	93.5	0.930	-	-
Date of surgery	100.0	99.4	4,916	86.4	0.840	-	-
Did you receive chemotherapy? (Yes/No)	100.0 (91.4% Yes)	100.0 (84.1% Yes)	4,948	90.1	0.550	98.3 (97.9-98.7)	45.2 (41.7-48.7)
Date started chemotherapy	91.2	83.6	4,068	79.0	0.790	-	-
Did you receive radiation therapy? (Yes/No)	100.0 (32.1% Yes)	100.0 (4.9% Yes)	4,948	71.7	0.170	88.9 (84.3-92.6)	70.9 (69.6-72.2)
Date radiation therapy started	32.1	4.93	217	79.3	0.790	-	-

^a ^For the purposes of comparison, all dates were either reported or recoded in YYYYMM format in both instruments.

^b ^PQ: Patient questionnaire.

^c ^MC: Medical chart.

^d ^Number of patients across both surveys that had data for a specific question.

### Accuracy of patient self-report regarding chemotherapeutic drug usage

Correct reporting of the use of the 10 most common chemotherapeutic drugs among this cohort of patients was evaluated and results are presented in Table 4. Fluorouracil, cyclophosphamide, epirubicin, methotrexate, calcium folinate, pirarubicin, a group of novel drugs, vinorelbine, docetaxel, and paclitaxel, in that order, were the 10 most commonly used drugs in our population according to both surveys (Table 4). Agreement ranged from 81.7% (epirubicin) to 98.0% (docetaxel) for these 10 agents; the most commonly reported agent, flurouracil, demonstrated high concordance (agreement: 84.9%, kappa: 0.64). Agreement tended to be higher for less commonly used drugs compared with more commonly used drugs, although the kappa statistics varied modestly. The self-reported usage rates for all ten drugs were consistently higher than rates obtained from medical charts. Again, this result is likely due to the longer time period covered by the self-reports.

**Table 4:** Concordance of Patient Recall with Medical Chart Data in Reporting Use of the 10 Most Commonly Prescribed Chemotherapeutic Agents for Breast Cancer Treatment, Shanghai, China, 2002-2006.

Chemotherapeutic Agent	% that reported use of drug on PQ^a^	% of MC^b ^that reported use of drug	Agreement (%)	Kappa
Fluorouracil	71.0	68.8	84.9	0.64
Cyclophosphamide	70.1	59.7	82.5	0.62
Epirubicin	52.4	46.0	81.7	0.64
Methotrexate	19.6	16.1	92.1	0.73
Calcium folinate	14.7	14.4	85.4	0.41
Pirarubicin	12.4	8.8	90.8	0.51
Novel drugs ^c^	9.5	4.7	95.7	0.63
Vinorelbine	8.1	6.9	96.7	0.76
Docetaxel	4.2	2.4	97.8	0.65
Paclitaxel	3.4	2.5	98.0	0.65

^a ^PQ: Patient questionnaire.

^b ^MC: Medical chart.

^c ^Novel drugs include the following list of pharmaceuticals, all of which were approved in China for use in breast cancer patients in 2002, the year both surveys were conducted: altretamine, oxaliplatin, gemcitabine, trastuzumab, ifosfamide, temozolomide, and Aredia.

Finally, we carried out regression analyses to evaluate whether socio-demographic factors, including age, time since diagnosis, stage of disease at diagnosis, education level, or income level, affected the concordance of patient recall with medical chart information in regards to both treatment-related and disease-parameter variables. We found no evidence that the concordance rates varied by these factors (data not shown).

## Discussion

Moderate to excellent concordance between self-reported and medical chart-based information was observed for both disease- and treatment-related variables. Patients accurately reported the dates of their diagnosis and associated surgical procedures, the location of the initial tumor, and ER/PR status. The reporting of various treatments, either chemotherapeutic drugs or otherwise, also demonstrated excellent agreement on most accounts, although the patient questionnaire invariably demonstrated higher usage rates for all forms of therapy, a finding likely attributable to the longer period of time covered by the patient survey relative to the medical chart. Levels of concordance did not vary according to demographic factors.

The high agreement values observed for treatment-related variables may, in part, be explained by the time-point at which study participants were interviewed. For example, breast cancer patients may still be undergoing adjuvant treatments at 6 months post-diagnosis, which may have aided in the recall of the treatment variables measured in our study. Our results are consistent with prior validation studies conducted among breast cancer cohorts in Iowa [16] and Quebec [17], particularly in regards to the high concordance observed with recall of treatment-related variables and dates of important disease-related events, such as the date of surgery or the initiation of chemotherapy. The recall of specific chemotherapeutic regimens among our Shanghai cohort was similar to results found in a previous validation study of 895 breast cancer patients in Melbourne, Australia [18], with agreement values ranging from 75% or greater for the most commonly prescribed regimens (cyclophosphamide, methotrexate, fluorouracil, doxorubicin, and epirubicin). In all of these studies, the time between patient interview and diagnosis was at least 1.5 years or greater; thus, the robustness of our self-report findings at only 6 months post-diagnosis necessitates future studies to examine significant changes in concordance over time.

The only known prior study that assessed the validity of self-reports among a disadvantaged or low-income population similar to our cohort was conducted by Liu et al. among 726 breast cancer patients aged 18 years or older living in California, two-thirds of whom had an average annual income of less than $20,000 [19]. Their study's results were remarkably similar to ours in that >98% of women were able to identify whether they had undergone surgery for breast cancer, >87% could identify the type of surgery conducted, >86% could report details of chemotherapeutic regimens, and the accuracy of recalling dates of diagnoses and surgeries was within 15 days of the dates documented in the medical records.

Other potential explanations for the high concordance values observed for most of our diagnosis- and treatment-related variables include the fact that hospital patients in Shanghai are given a summary record of information pertinent to their hospital visits upon discharge. This information includes relevant diagnoses, lab results, treatments pursued, and the follow-up plan for future care. This information is succinct, in layman's terms, and can be brought by the patient to any hospital establishment that they visit for their care. However, there is no reliable metric of how often patients bring this informal hospital discharge summary to future doctor appointments or emergency department visits. In addition, given the emphasis on family and community in Chinese culture, many patients are accompanied by family members or close friends to the hospital. This may assist patients with recall, and in general, having a family member cued into a patient's care could certainly help explain our results.

The low concordance values observed for information related to radiotherapy may best be explained by the difference in the exposure window covered by each assessment tool and by use of different care providers. Whereas adjuvant chemotherapy and curative surgery are common first-line interventions instituted soon after diagnosis (and hence, likely recorded by the diagnosing hospital's medical chart), radiotherapy may only be used in advanced cases of disease for palliative purposes or in the event that surgery with chemotherapy did not control metastatic spread. We see evidence of this in the differences in numbers between medical chart-abstraction data and patient self-reports. For example, according to medical chart data only 4.93% (n = 244) of patients received radiotherapy compared with 32.1% (n = 1,588) according to patient self-reports. Therefore, the wide gap in rates of report for radiotherapy across both surveys may be a result of the algorithmic approach to breast cancer treatment in our cohort: second- and third-line treatment options that are not employed immediately after diagnosis were not captured by the initial medical chart review. A similar problem in relation to comparing drug usage across surveys conducted at different points in time has been previously described in detail [2] and represents a limitation of our study.

Our population-based study design and high response rate enhance the generalizability of our findings, and this represents a primary strength of our study. The structured questionnaires that were used in the survey and medical chart review helped to reduce misclassifications and errors during data abstraction. Given that multiple diagnostic hospitals were involved in the study, in addition to the lack of standardization in the recording of medical chart information for research purposes, misclassifications when analyzing the medical chart data are likely. Therefore, the agreement rates that we observed in our study are likely to be underestimated.

## Conclusion

In summary, our study showed excellent concordance in the reporting of essential disease- and treatment-related information between the medical chart and patient self-report among breast cancer patients 6 months after diagnosis. Among our most important findings was breast cancer patients' ability to recall the use of specific chemotherapeutic drugs, in addition to various diagnostic characteristics of their disease. Taken together, our results have significant implications for conducting population-based studies among breast cancer patients, as the collection of medical information via hospital records can be both cost-prohibitive and logistically complicated, because of concerns regarding patient confidentiality. While our results are not necessarily applicable to populations globally, in settings where patients seek medical care at multiple hospitals for practical or financial considerations, as is often the case in China, our findings reinforce the utility of patient self-report as an accurate means of obtaining medical data to guide data collection and clinical decision-making.

## Abbreviations

ER: estrogen receptor; IQR: inter-quartile range; PR: progesterone receptor; SBCSS: Shanghai Breast Cancer Survival Study; TNM: tumor node metastasis.

## Competing interests

The authors declare that they have no competing interests.

## Authors' contributions

VG conducted data analysis and interpretation, in addition to primarily drafting the paper and addressing revisions. GK, ZC, and WL assisted with the design of the study, implementation, and data analysis. XOS and YZ designed the study, directed the data analysis, and assisted with draft preparation. All authors read and approved the final manuscript.

## Pre-publication history

The pre-publication history for this paper can be accessed here:

http://www.biomedcentral.com/1471-2288/11/72/prepub
